# The immunological landscape of CCL26^High^ invasive oral squamous cell carcinoma

**DOI:** 10.3389/fcell.2025.1502073

**Published:** 2025-01-27

**Authors:** Lingyun Liu, Shuo Guan, Yizhuo Xue, Yijia He, Liang Ding, Yong Fu, Sheng Chen, Zhiyong Wang, Yi Wang

**Affiliations:** ^1^ Central Laboratory of Stomatology, Affiliated Hospital of Medical School, Nanjing Stomatological Hospital, Nanjing University, Nanjing, China; ^2^ Nanjing Stomatological Hospital, Affiliated Hospital of Medical School, Institute of Stomatology, Nanjing University, Nanjing, China; ^3^ Department of Oral and Maxillofacial Surgery, Affiliated Hospital of Medical School, Nanjing Stomatological Hospital, Nanjing University, Nanjing, China; ^4^ Department of Oral Pathology, Nanjing Stomatological Hospital, Medical School of Nanjing University, Nanjing, Jiangsu, China

**Keywords:** OSCC, CCL26, survival prognosis model, immune cell, immune check points, immunotherapy

## Abstract

**Background:**

Our previous study demonstrated that CCL26 secreted by cancer-associated fibroblasts (CAF) promoted the invasive phenotype of oral squamous cell carcinoma (OSCC), however, more comprehensive clinical expression patterns of CCL26 and its role in immunotherapy remains ambiguous.

**Methods:**

CCL26 levels in different cancer and normal tissues were analyzed and validated in 67 OSCC patients through immunohistochemical staining (IHC). The clinical spatial distribution pattern of CCL26 in tumor microenvironment was determined, and its clinical outcomes were investigated. We also determined the invasive phenotype of tumor cells with distinct CCL26 level and explored its immune checkpoint and immunocytes relevance by differentially expressed gene (DEG) analysis, GSEA, and GO analysis. We collected peripheral blood from 28 OSCC patients to assess the percentage and absolute number of lymphocytes by flow cytometry.

**Results:**

CCL26 was upregulated in HNSC and preferentially high-expressed on CAFs and tumor cells in OSCC patients, which exhibits a trend toward decreased overall survival. CCL26^high^ OSCC had a characteristic of tumor invasive phenotype with upregulated CLDN8/20 and reduced keratin KRT36, which was significantly associated with EMT markers (CDH1, CDH2, VIM, SNAI2). In addition, CCL26^high^ OSCC was found to be associated with immunoglobulin mediated immune response, B cell mediated immunity et al. Indeed, immune checkpoint molecules (PD-L1, PD-L2, et al.) also decreased in CCL26^high^ OSCC. However, CCL26 did not affect T/B/NK lymphocytes in peripheral blood of OSCC patients.

**Conclusion:**

CCL26 could regulate Immune balance and promote invasiveness of OSCC, which gave a new insight into a potential immunotherapy strategy.

## Introduction

OSCC, accounting for over 90% of all oral cancers, is a major global public health issue with minimal improvement in prognosis over the last three decades (Shrestha et al., 2020; Johnson et al., 2020). This highly heterogeneous cancer is marked by local invasion and immune suppression (Hung et al., 2024; Estephan et al., 2024; Yang et al., 2021), significantly impacting prognosis at molecular and histological levels (Liu et al., 2024). It has long been shown that different pattern of invasion (POI) has varying invasive capacities (Bryne et al., 1989; Brandwein-Gensler et al., 2005; Rivera-Colon et al., 2020; Morales-Oyarvide and Mino-Kenudson, 2016; Langner et al., 2006). In more aggressive forms, major pro-inflammatory cytokines and chemokines drive tumor progression (Mamun et al., 2022; Kondoh and Mizuno-Kamiya, 2022; Do et al., 2020; Tokunaga et al., 2020; Chen et al., 2015). Our prior research showed that CCL26 secreted by CAF in the worst pattern of invasion (WPOI) type 4–5 alters the tumor phenotype and correlates with reduced patient survival (Ding et al., 2022). Current literature on CCL26’s role in OSCC, beyond our studies, remains scant.

CCL26, also known as eosinophil chemokine-3, is expressed mainly by macrophages and epithelial cells and has chemotactic effects on eosinophils, monocytes and MDSC (Korbecki et al., 2020). It acts by binding to CX3CR1. Regenerated liver phosphatase-3 has been found to promote colorectal cancer invasion and metastasis by inducing TAMs infiltration through upregulation of CCL26 (Lan et al., 2018). Although previous study has shown that CCL26 is frequently dysregulated to promote the onset and progression of many malignancies (Kawano et al., 2021; Donlon et al., 2020; Luo et al., 2018), research to prove their validity in OSCC was lacking, which is crucial for future studies of CCL26-targeted therapy for OSCC. At the same time, CCL26 was previously shown to bind to and activate CCR3, a chemokine-receptor pair that may play an important role in a range of immune-mediated diseases such as persistent asthma (Larose et al., 2015), CCL26 has been shown to be the most potent inducer of eosinophil migration, and increasing evidence suggests that aberrant CCL26 plays a role not only in influencing tumor invasion, but also in shaping alterations in the tumor immune microenvironment (TIME) infiltration (Domaingo et al., 2023), ultimately impacting the efficacy of immunotherapy (Li et al., 2023; Heeran et al., 2021; Hu et al., 2021; Tudoran et al., 2015). While previous studies have offered preliminary insights into the role of CCL26 in specific cancers, targeting chemokines and their receptors has been proposed as a promising strategy for immunotherapy (Qin et al., 2023), its broader implications in the realm of immunotherapy across OSCC remain unknown. Tumor immunotherapy is a relatively novel therapeutic approach that holds promise for controlling tumor recurrence and metastasis (Chen et al., 2023). Currently, immunotherapy options for OSCC are extremely limited, so we urgently need more therapeutic targets to improve the survival and prognosis of OSCC patients.

**FIGURE 1:**
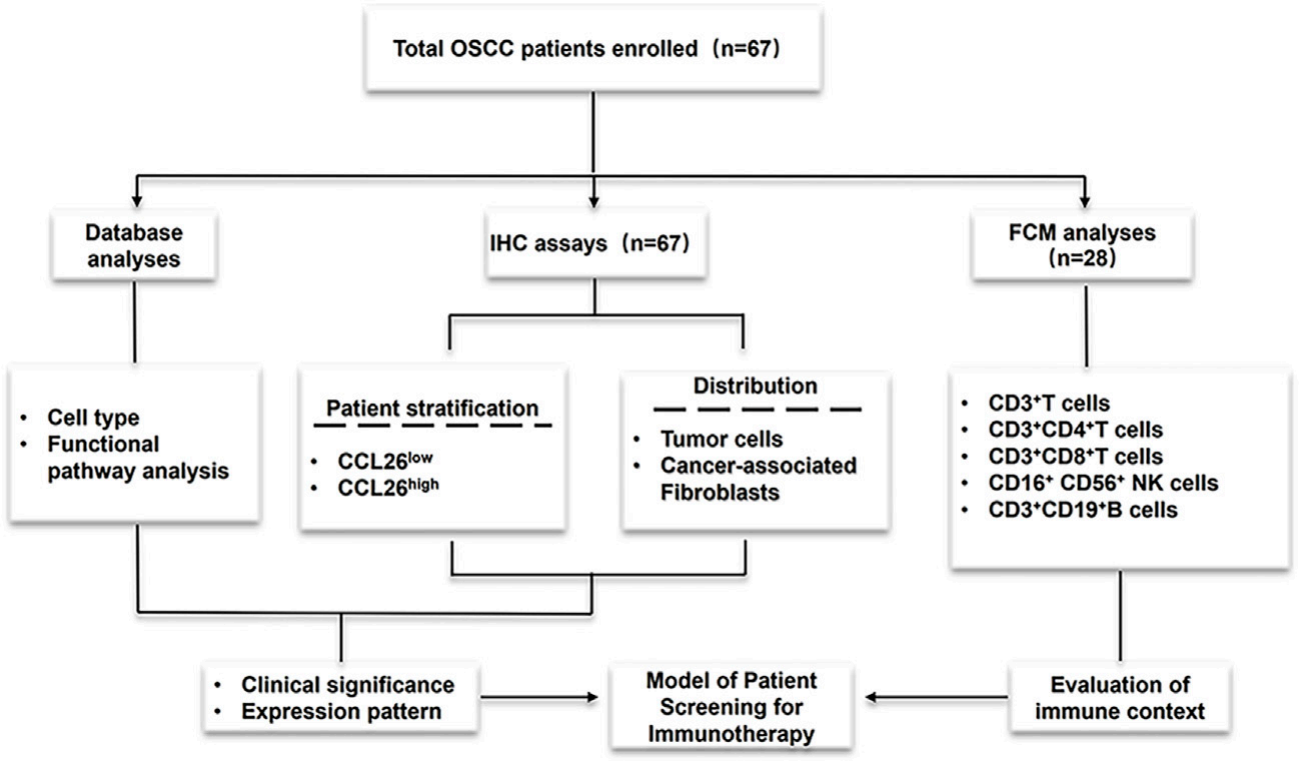
Experimental process design diagram Cohort studies were conducted through three methods (database analyses and IHC assays to evaluate CCL26 clinical expression and prognosis; Flow Cytometry to assess immune context) and flowchart for characterization of the patients enrolled in this study.

Therefore, in this work, we systematically investigated the clinical expression patterns, clinicopathological features, and prognostic value of CCL26 chemokines in the tumor microenvironment (TME). The spatial distribution pattern of CCL26 in OSCC was elucidated, and it was preliminarily confirmed that it was closely associated with poor patient prognosis; moreover, we used bioinformatic analysis methods such as multiple tumor databases and gene enrichment in order to reveal its potential functional mechanisms. Preliminary findings suggest that it may be associated with altered invasive phenotype and tumor immunosuppressive microenvironment, and importantly, the relationship between CCL26 and immune homeostasis and immune checkpoint pathway was also examined. Our findings may provide some new insights into CCL26 as a potential molecular marker in OSCC treatment, especially its therapeutic potential in immunotherapy, laying the foundation for further functional experiments in the future. The research concept of this paper is shown in Figure 1.

## Methods

### Patients and samples

We retrospectively collected data from 67 primary OSCC patients at various stages (I-IV) who underwent radical resection at Nanjing Stomatological Hospital between 2010 and 2017. The ethics committee of the Affiliated Nanjing Stomatological Hospital of Nanjing University Medical School approved all experiments. Informed consent was obtained for the use of patient tissues and data, following the Helsinki Declaration. Formalin-fixed, paraffin-embedded OSCC tissues were used for IHC. Patients who had received preoperative treatments or had systemic diseases or incomplete data were excluded. Of these, 28 had both postoperative tumor and preoperative blood samples, while 39 had only tumor samples. Next, based on the IHC results of the 67 patients, the clinical expression pattern of CCL26 was evaluated. More detailed patient inclusion and exclusion criteria can be found in our previous study (Zhu et al., 2020). The clinical characteristics of the enrolled patients are shown in Table 1.

**TABLE 1:** Clinical characteristics of enrolled patients.

All cases	Total	N (%)
	67	100.00%
Gender		
Male	48	71.70%
Female	19	28.30%
Age		
≤60	27	40.30%
>60	40	59.70%
Smoking		
Yes	23	34.30%
No	44	65.70%
TNM		
Ⅰ-Ⅱ	26	38.80%
Ⅲ-Ⅳ	41	61.20%
T-stage		
1–2	38	56.70%
3–4	29	43.30%
Lymph node metastasis		
No	38	56.70%
Yes	29	43.30%

### IHC and quantification

IHC was performed as previously described, incubating sequential sections with primary antibodies such as anti-CCL26(ab217328, Abcam). The IHC staining results for CCL26 were independently evaluated by two senior pathologists and the average values were calculated for further analysis. IHC staining was scored based on the percentage of positive cells and staining intensity. The percentage of stained cells was defined as: 0 = 0–5%; 1 = 6–25%; 2 = 26–50%; 3 = 51–75%; 4 = 75–100%. Staining intensity was defined as follows: 0 = negative staining; 1 = weak staining; 2 = moderate staining; 3 = strong staining. The staining intensity score was multiplied by the percentage of positive cells to calculate the IHC score. High and low expression of CCL26 was defined based on the median IHC score.

### Flow cytometry

Peripheral blood mononuclear cells (PBMCs) were collected from the preoperative whole blood of patients. To analyze PBMC subtypes, cells were washed twice with phosphate-buffered saline (PBS, Servicebio, Wuhan, China) and resuspended in 200 μL PBS. BD Multitest™ reagents were used to count CD3^+^ T cells, CD3^+^ CD4^+^ T cells, CD3^+^ CD8^+^ T cells, CD19^+^ B cells, and CD56^+^ NK cells. Quantification was performed using a fluorescence-activated cell sorting (FACS) Calibur instrument. All participants in the study gave informed consent.

### Gene correlation analysis in cBioPortal

cBioPortal for Cancer Genomics (http://cbioportal.org) is a website for exploring multidimensional cancer genomics data. We used cBioPortal to analyze the correlation between CCL26 and specific immune cell subset markers and immune checkpoint molecules in head and neck squamous cell carcinoma (HNSCC). Co-expression was calculated according to the online instructions of cBioPortal.

### Public data download and processing

Gene expression data and clinical information of HNSCC patients from the TCGA database were downloaded using the R package TCGA biolinks, according to the norms of pathologic diagnosis of oral cancer, excluding non-OSCC patient samples (originate from the epithelium of the oropharyngeal mucosa, including the mucosa of the soft palate, the base of the tongue (the posterior 1/3 of the sulcus), the lateral pharyngeal). Gene Set Enrichment Analysis (GSEA) was performed using the R package cluster Profiler: first, the expression levels of CCL26 were classified into high and low expression groups, and the log_2_FC of genes between the high and low expression groups was calculated using the DESeq2. Then, GSEA was performed with log_2_FC as the enrichment weight. The gene sets were downloaded from the gene set in the MsigDB database.

### Single-cell RNA sequencing analysis in TISCH2

Tumor Immune Single-Cell Hub 2 (TISCH2) is a resource of single-cell RNA-seq data from human and mouse tumors that comprehensively describes the gene expression of the TME in various cancer types. Single-cell sequencing of HNSC_GSE103322 was used to validate the expression pattern of CCL26 in head and neck tumors and its relationship with clinical staging.

### Statistical analysis

Data analysis and graphical processing were performed using SPSS 26.0(IBM Corp, Armonk, NY, United States), GraphPad Prism 10.0 (Dotmatics, Boston, MA, United States), and Chiplot (https://www.chiplot.online/). Pearson’s chi-square test, Fisher’s exact test, and chi-square test were used to compare clinicopathological features. The Mann-Whitney U test was used to compare two patient groups. Survival analysis, including overall survival (OS), metastasis-free survival (MFS), and disease-free survival (DFS), was assessed using the Kaplan-Meier test and log-rank test. The Cox proportional hazards regression model was further used for multivariable analysis to determine independent risk factors for OSCC, adjusting hazard ratios (HRs) and 95% confidence intervals (CIs). Pearson correlation analysis was used to study the co-expression of CCL26 immune cell markers and immune checkpoint molecules. All statistical tests were two-sided, and *P* < 0.05 was considered statistically significant.

## Results

### CCL26 was expressed in the TCs and CAFs within the OSCC tumor microenvironment

**FIGURE 2:**
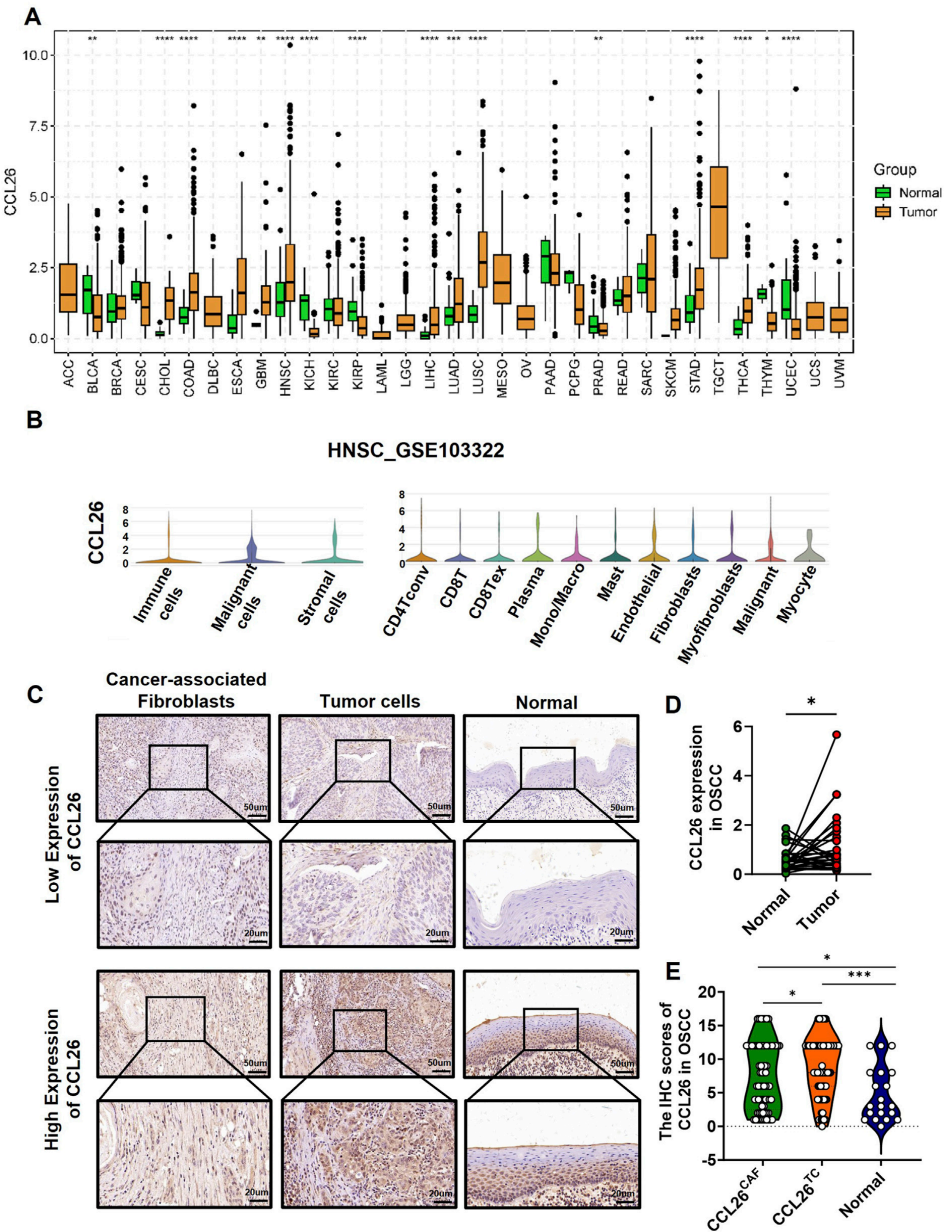
Expression pattern of CCL26 in OSCC and other tumors. **(A)** The CCL26 expression in different tumor types using TCGA database. **(B)** Expression of CCL26 in HNSCC was analyzed by single-cell RNA sequencing (GSE103322). **(C)** Typical IHC staining of CCL26 on TCs, CAFs and normal tissue. **(D)** RNA-seq data from TCGA study showed upregulation of CCL26 in OSCC as compared with adjacent normal tissues (paired and unpaired samples, *n* = 30). **(E)** The IHC score of CCL26 in TCs, CAFs (n = 67) and normal tissue (n = 30) from OSCC patients. TCs, tumor cells; CAFs, cancer-associated fibroblasts. *, **, *** and ****represents that differences were considered statistically significant with *p* < 0.05, *p* < 0.01, *p* < 0.001 and *p* < 0.0001.

Using the TCGA database, we analyzed CCL26 mRNA levels across various normal and corresponding tumor tissues (Figure 2A), and found that the mRNA expression of CCL26 expression was abnormally high in several cancers compared to normal tissues, particularly in cholangiocarcinoma, colorectal, esophageal squamous, head and neck squamous, hepatocellular, lung, and gastric carcinomas and it was markedly downregulated in bladder uroepithelial, renal, and gastric cancers. Notably, in head and neck cancers, CCL26 expression was 4–5 times higher in tumor tissues than in normal counterparts.

Single-cell sequencing from the TISCH2 database showed CCL26’s broad distribution across various cell types, including tumor cells, mesenchymal stromal cells, and immune cells in head and neck cancers (Figure 2B) (GSE103322). *In situ* immunohistochemistry of OSCC samples revealed widespread CCL26 expression in both tumor cells and CAFs, localized to the cell membrane and cytoplasm (Figure 2C). Notably, expression levels were higher in tumor cells (Figure 2D), consistent with our previous findings on CAF-derived CCL26’s role in tumor invasiveness.

### Upregulated of CCL26 negatively correlated with poor prognosis of OSCC

**FIGURE 3:**
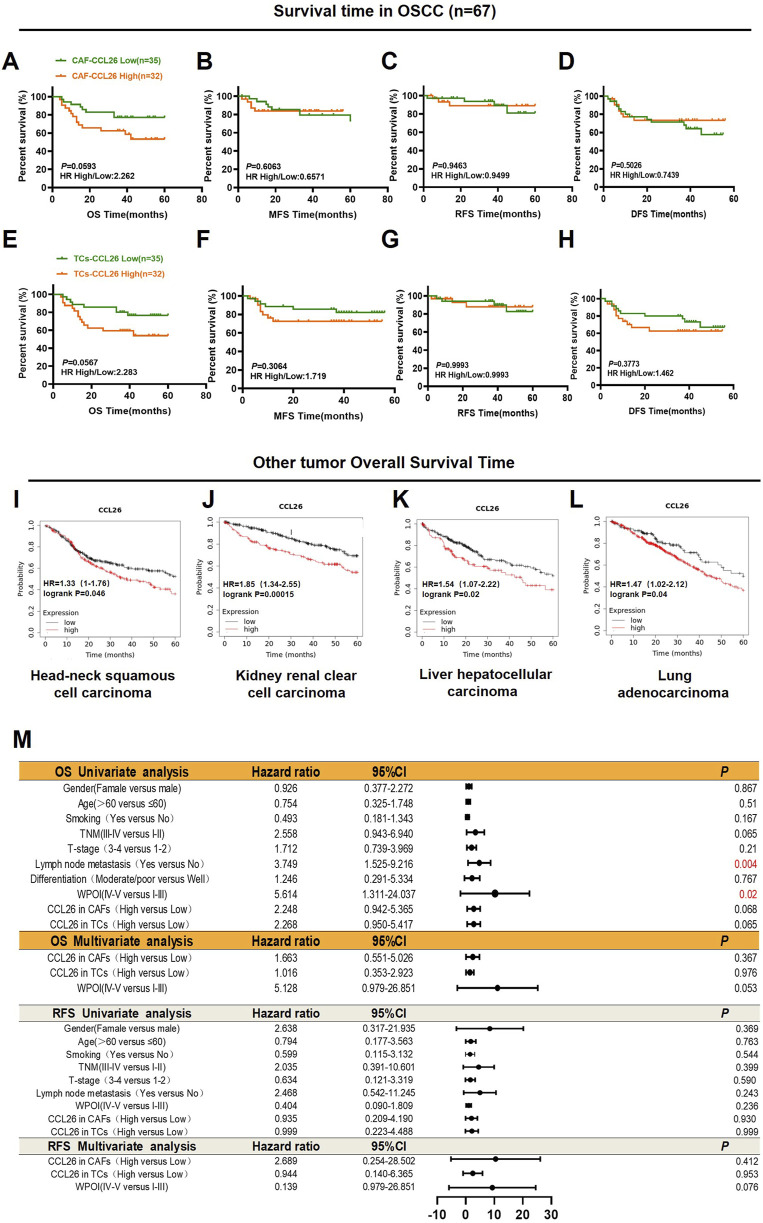
CCL26 heterogeneity in tumor microenvironment leads to different clinical outcomes. **(A–H)** Kaplan–Meier survival analyses for overall survival time (OS), metastasis-free survival time (MFS), recurrence-free survival (RFS) and disease-free survival time (DFS) of OSCC patients according to the protein expression of CCL26 in TCs, CAFs. **(I–L)** The effects of CCL26 expression on the prognosis of OS in Head-neck squamous cell carcinoma, Kidney renal clear cell carcinoma, liver hepatocellular carcinoma and Lung adenocarcinoma patients were shown by Kaplan–Meier plotter database. **(M)** Cox regression models for OS and RFS in OSCC patients to determine the independent risk factors, adjusted hazard ratio (HR), and 95% confidence interval (CI) of OSCC. Survival analyses including OS, MFS, RFS, and DFS were evaluated by Kaplan–Meier and log-rank test.

We analyzed the association between CCL26 expression in TCs and CAFs from *in situ* tissues and the 5-year postoperative outcomes (overall survival, recurrence, and metastasis) for 67 patients. Although high CCL26 expression in TCs and CAFs trended towards poorer overall survival, this correlation was not statistically significant (*P* > 0.05) (Figures 3A, E). Additionally, there was no significant association between increased CCL26 expression and postoperative recurrence or metastasis risks (*P* > 0.1) (Figures 3B–D, F–H). The relationship between CCL26 expression and 5-year overall survival in other types of tumors was analyzed through the Kaplan-Meier plotter database (http://kmplot.com/analysis/index.php?p=service), and similar results were observed, with patients whose tumor cells highly expressed CCL26 having significantly shorter 5-year overall survival in head and neck squamous carcinomas, renal clear cell carcinomas, hepatocellular carcinomas, and lung carcinomas (Figures 3I–L).

To analyze the prognostic value of different clinicopathological features, we used univariate and multivariate Cox regression analysis. Our data confirmed that WPOI and lymph node metastasis in OSCC were associated with shorter overall survival in univariate analysis, whereas no prognostic factors significantly affecting OSCC have been observed in multivariate models (Figure 3M). Notably in CAFs CCL26 expression was significantly associated with age, degree of differentiation, and WPOI (*P* < 0.05) whereas in TCs with smoking and WPOI (*P* < 0.05) (Table 2). These results suggest that CCL26 may be a potential poor prognostic factor for OSCC, but not an independent prognostic factor.

**TABLE 2:** Association between CCL26 expression and clinicopathological characteristics in OSCC patients.

Characteristics	N	CCL26 protein expression in CAFs				N	CCL26 protein expression in TCs			
		Low n (%)	High n (%)	*X* ^2^	*P*		Low n (%)	High n (%)	*X* ^2^	*P*
Sex	67					67				
Male		28 (41.8)	20 (29.9)	2.52	0.112		23 (34.3)	25 (37.3)	1.267	0.26
Female		7 (10.4)	12 (17.9)				12 (17.9)	7 (10.4)		
Age (years)	67					67				
<60		19 (28.4)	8 (11.9)	5.959	0.015*		11 (16.4)	16 (23.9)	2.396	0.122
≥60		16 (23.9)	24 (35.8)				24 (35.8)	16 (23.9)		
Smoking	67					67				
Yes		11 (16.4)	12 (17.9)	0.273	0.601		18 (26.9)	5 (7.5)	9.505	0.002*
No		24 (35.8)	20 (29.9)				17 (25.4)	27 (40.3)		
TNM	67					67				
I-II		13 (19.4)	13 (19.4)	0.085	0.77		14 (20.9)	12 (17.9)	0.044	0.834
III-IV		22 (32.8)	19 (28.4)				21 (31.3)	20 (29.9)		
T stage	67					67				
1–2		17 (25.4)	21 (31.3)	1.98	0.159		21 (31.3)	17 (25.4)	0.322	0.57
3–4		18 (26.9)	11 (16.4)				14 (20.9)	15 (22.4)		
Lymph node metastasis	67					67				
No		22 (32.8)	16 (23.9)	1.126	0.289		22 (32.8)	16 (23.9)	1.126	0.289
Yes		13 (19.4)	16 (23.9)				13 (19.4)	16 (23.9)		
Differentiation	67					67				
Well		7 (10.4)	0 (0)	5.169	0.023*		3 (0.04)	4 (0.06)	0.016	0.9
Moderate to poor		28 (41.8)	32 (47.8)				32 (47.8)	28 (41.8)		
WOPI	67					67				
I-III		18 (26.9)	3 (0.04)	13.738	<0.001		15 (22.4)	6 (9.0)	4.514	0.034*
IV-V		17 (25.4)	29 (43.3)				20 (29.9)	26 (38.8)		

### CCL26 promotes an altered invasive phenotype through EMT in TCs

**FIGURE 4:**
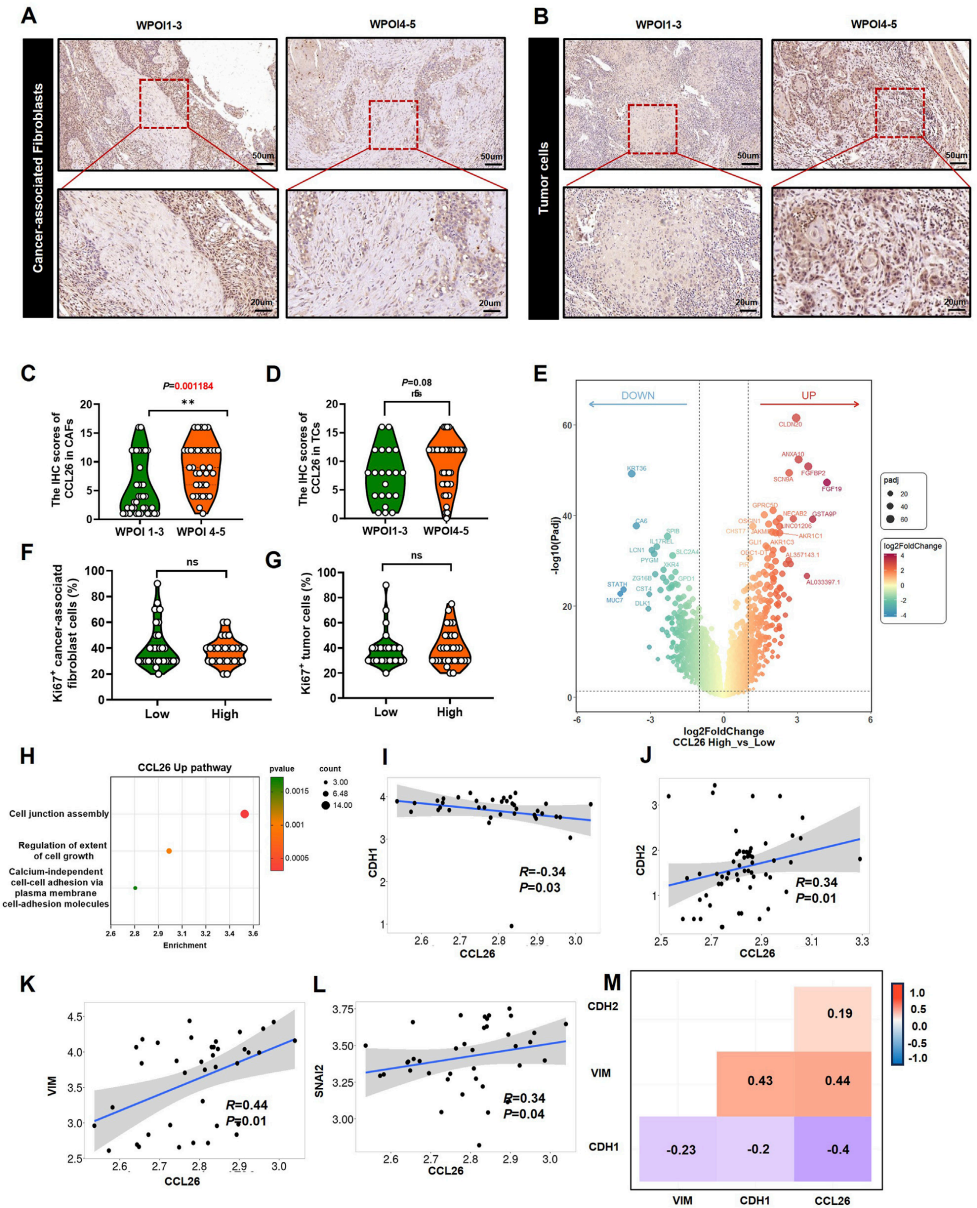
CCL26 is associated with the invasive phenotype of TCs. **(A, B)** CCL26 with different WPOI in CAFs and TCs. **(C, D)** The IHC score of CCL26 in different WPOI of TCs, CAFs from OSCC patients, n = 67. **(E)** DEG analysis for differently expression mRNAs in OSCC tissues with CCL26 expression in TCGA datasets. **(F, G)** Correlation between Ki-67 staining and CCL26 in TCs and CAFs. **(H)** GO annotated CCL26^high^ mediated pathway. **(I–M)** Correlation between CCL26 RNA expression and CDH1, CDH2, VIM and SNAI2 in tumor of oral cavity from TNMplot.

In view of CCL26 expression in TC and CAF was significantly associated with WPOI and that patient OS was affected by WPOI. We performed an *in-situ* analysis of the relationship between CCL26 expression and WPOI in these two cell types (Figures 4A, B). The results showed that CCL26^high^ in CAFs was significantly correlated with WPOI (*P* < 0.01), whereas in TC, CCL26^high^ indicated a tendency for worsening of the invasion pattern (*P* = 0.085) (Figures 4C, D). Interestingly, OSCC patients with CCL26^high^ tumor cells and CAFs did not show higher Ki-67 positivity (*P* > 0.05) (Figures 4F, G), and we hypothesized that CCL26 had no direct effect on the growth of tumor cells and CAFs.

To explore the more comprehensive effect of CCL26 on OSCC, we performed transcriptome analysis of differentially expressed genes in tissue samples with high and low CCL26 expression using database information (Figure 4E). We excluded non-OSCC samples from the HNSCC data downloaded from the TCGA database. The analysis showed that high expression of CCL26 downregulated KRT36, which is a keratin family member previously thought to be inactivated during tongue tumorigenesis (Brychtova et al., 2020), resulting in the loss of epithelial features of tumor cells, and closely associated with the onset of the EMT process (Lamouille et al., 2014). At the same time, CLDN20, CLDN8 and other CLDN family genes are upregulated, which regulate intercellular tight junctions (Figure 4H), and the tightly packed epithelial cells first weaken or depolymerize intercellular junctions one after another in the process of EMT (Adil et al., 2021), and the cell morphology eventually changes from epithelial-like to mesenchymal-like. Wang W et al. reported that the CLDN family is closely related to EMT in hepatocellular cancer (Wang et al., 2024), as well, Chang JW et al. show that CLDN1 promoted invasive phenotypes by upregulating epithelial-to-mesenchymal transition (EMT) in HNSC (Chang et al., 2022), although the relationship between CLDN20, CLDN8, *etc.*, and EMT is poorly reported. After we performed enrichment pathway analysis of differential genes, we also found that cell junctional assembly pathways were significantly enriched in the CCL26^high^ group; these findings suggest that CCL26 has the potential to affect tumor cells by regulating EMT. Further database exploration revealed that in oral and tongue tumors (TNMplot), we found that CCL26 had a significant correlation phenotype with four classical markers of EMT: among them, it was negatively correlated with E-cad (R = −0.34, *P* < 0,05), and significantly correlated with N-cad (R = 0.34, *P* < 0,05), VIM (R = 0.44, *P* < 0,05) and SNAI2 (R = 0.34, *P* < 0,05) were positively correlated (Figures 4I–M), suggesting that CCL26 may affect the invasive phenotype of oral cancer cells through mesenchymal transformation.

### Immunological implications of CCL26 within the tumor microenvironment

**FIGURE 5:**
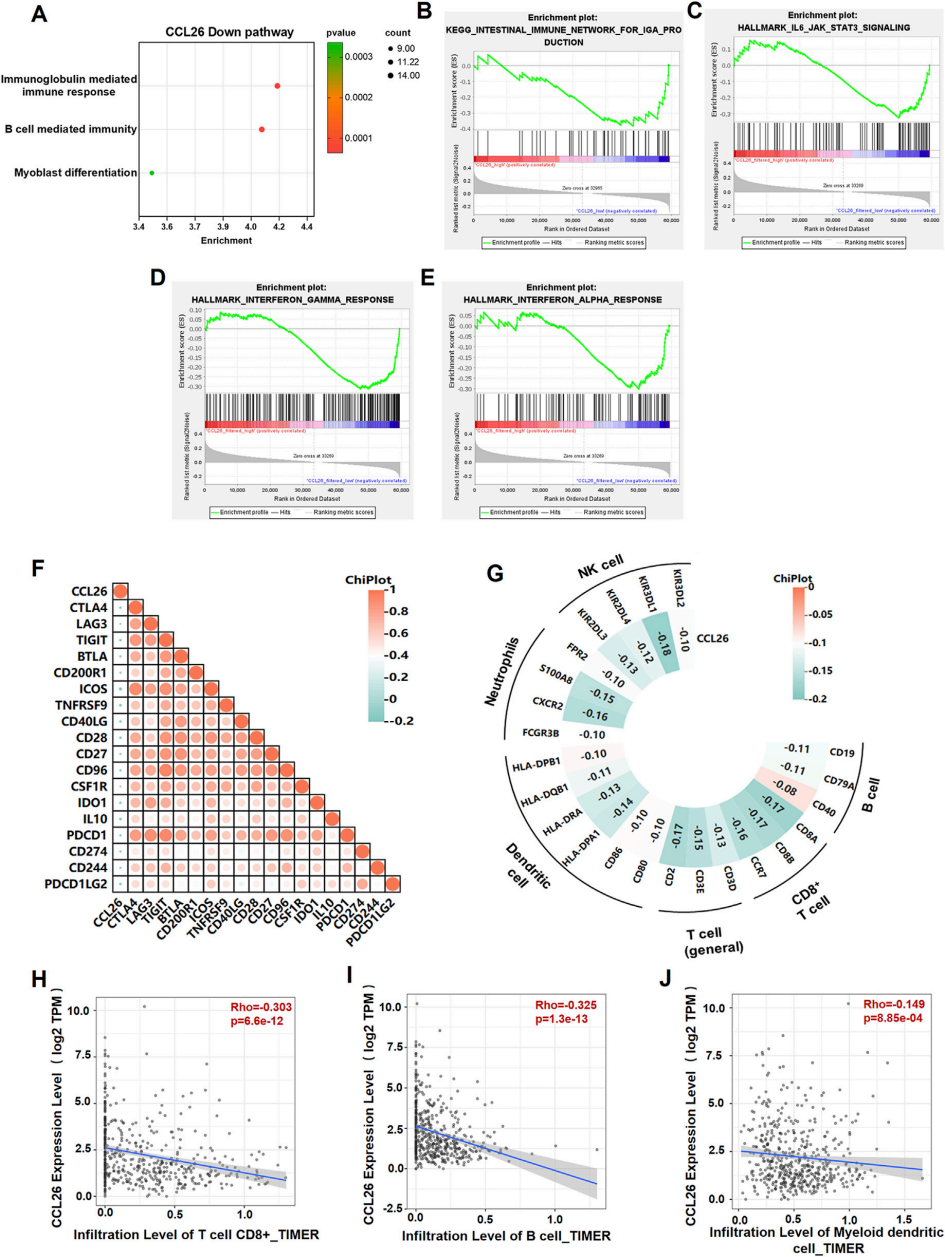
CCL26 is associated with the immunosuppressive microenvironment of tumor tissue. **(A)** GO annotated CCL26^low^ mediated pathway. **(B–E)** GSEA analysis for differently expression mRNAs in OSCC tissues with CCL26 expression in TCGA datasets. **(F)** Heatmap illustrating the correlation between CCL26 and immune checkpoints from cBioportal database. **(G–J)** Correlation analysis between CCL26 and immune cell infiltrations in HNSCC samples using TIMER and cBioportal. Results are shown by Pearson correlation analysis.

Immunotherapy has ushered in a new era of cancer treatment, and cancer immunotherapy continues to be revitalized. In recent years, Zhao et al. obtained a 7 immune-related genes prognostic model for OSCC, including CGB8, CTLA4, TNFRSF19, CCL26, NRG1, TPM2 and PLAU), provided a promising biomarker and a way to monitor the long-term treatment of OSCC (Zhao et al., 2021). To reveal the relationship between CCL26 and immunotherapy resistance, we analyzed various immune aspects of CCL26 in TIME. We excluded the non-OSCC sample in the HNSCC data (downloaded from the TCGA database), and then performed GSEA and Go analyze on these data to understand the molecular basis of the oncogenic property and identify the potential signaling pathways involved in CCL26 expression. Several immune-related pathways were enriched in the CCL26^low^ group (immunoglobulin mediated immune response, B cell mediated immunity) (Figures 5A–E), suggests that CCL26 may play a role in suppressing tumor immunity. Furthermore, we performed TIMER (http://timer.cistrome.org/) algorithms to quantify the relationship between CCL26 expression and multiple immune cell infiltrations (Figures 5H–J). We found that the expression of CCL26 tended to correlate negatively with the infiltration of CD8^+^ T cells in OSCC, and also negatively correlated with the infiltration of other immune cells such as B cell transformation (Figure 5G). In general, a relatively high CD8^+^ T-cell infiltration in the tumor microenvironment is often defined as a hot tumor and *vice versa* as a cold tumor. These results further demonstrate that CCL26 is involved in modulating the immune microenvironment of cold tumors and may have an inhibitory role in the anti-tumor immune process.

Considering the robust correlation with immune cells, we explored CCL26’s association with immunomodulatory molecules, including the B7-CD28 family, tumor necrosis factors (TNF) family, and other classic and novel immune checkpoints. The results demonstrated that these immune checkpoints are closely related to CCL26 expression in different cancers, namely CTLA4, LAG3, PDCD1, CD28, CD274, and TIGIT et al. exhibited a negative association with CCL26 in HNSC (Figure 5F). Most of these inhibitors have been identified as key effectors in the response to immunotherapy and novel immunotherapeutic targets, suggesting that the target CCL26 may benefit less from immunotherapy. It is also reasonable to speculate that CCL26 acts an indispensable function in the response to tumor immunotherapy.

### CCL26^high^ tumor showed on impacts on the peripheral circulating lymphocytes

**FIGURE 6:**
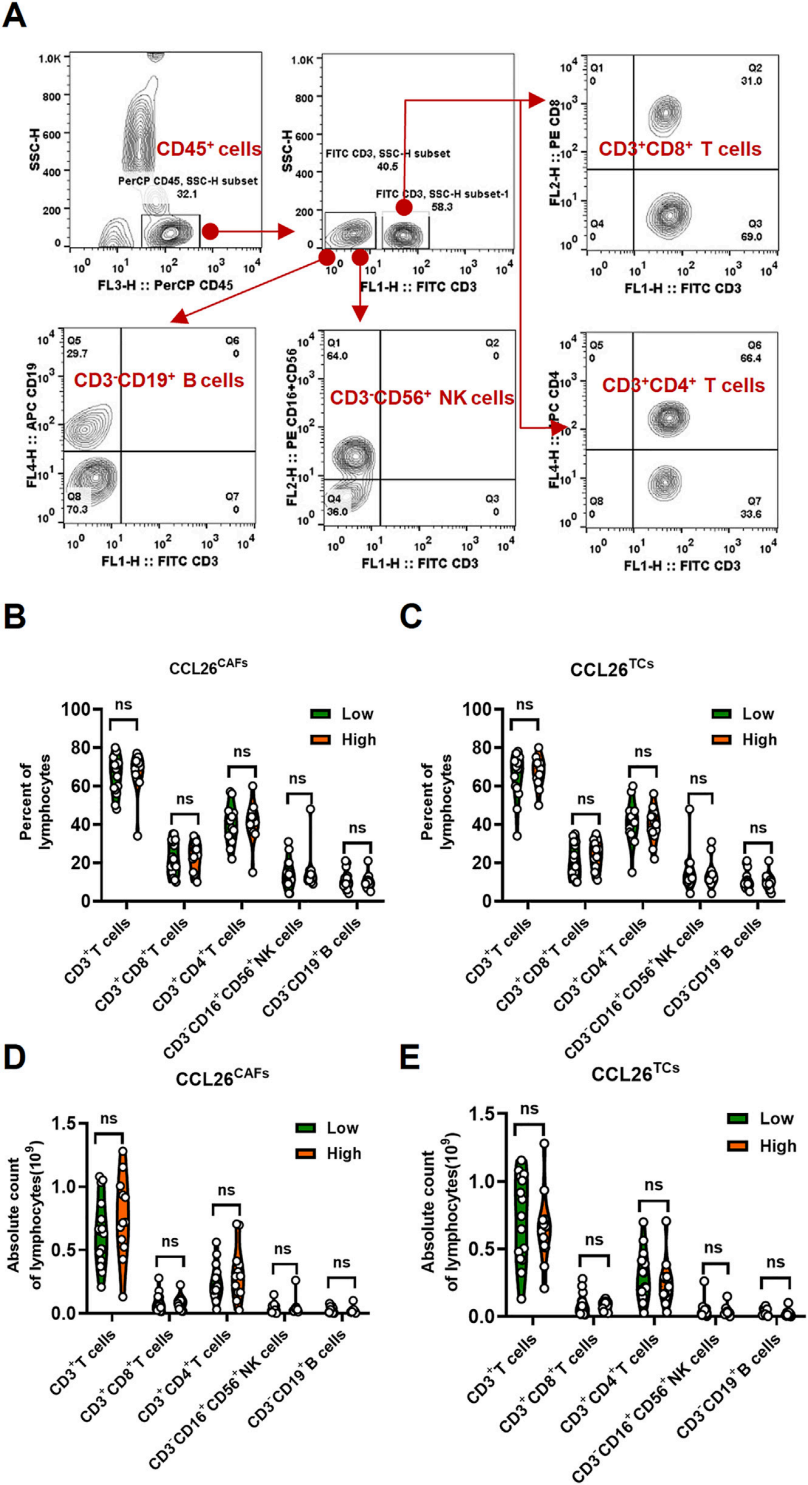
The change of lymphocytes subset in PBMC of OSCC patients according to CCL26 level. **(A)** Flow cytometry contour plots showing the strategy for gating lymphocytes. **(B–E)** The ratio and absolute number of human CD3^+^T cells, CD3^+^CD4^+^ helper/inducer T cells, CD3^+^CD8^+^ cytotoxic T cells, CD3^−^CD16^+^CD56^+^ NK cells and CD3^−^CD19^+^ B cells in blood were analyzed in CCL26^low^ and CCL26^high^ groups of TCs and CAFs by BD Multitest™ reagent in OSCC patients (n = 28). Results are shown by two-way ANOVA. p = Sidak’s multiple comparison test. ns, no significance.

Since tumor metastasis requires cancer cells to circulate in the bloodstream, withstand pressure in the blood vessels, and evade deadly battles with immune cells (Ganesh and Massagué, 2021; Fares et al., 2020). The clinical goal of cancer immunotherapy is to stimulate the host’s immune system to develop passive or active immunity against malignant tumors. Meanwhile, immune cells are the cellular basis of cancer immunotherapy. Chiu DK et al. reported that MDSCs preferentially infiltrate into hypoxic regions of human hepatocellular carcinoma tissue, and hypoxia-induced MDSC infiltration is dependent on hypoxia-inducible factors, hypoxia-inducible factor activates the transcription of CCL26 in cancer cells, which recruits MDSC expressing the chemokine CX3CR1 to primary tumors, significantly increasing angiogenesis and promoting tumor growth (Chiu et al., 2016). Hence, to investigate the role of CCL26 in OSCC on tumor immunity, we next analyzed the ratio and absolute number of key immunocytes in peripheral blood of OSCC patients (n = 28) by flow cytometry (CCL26^high^ and CCL26^low^ groups):CD3^+^ T cells, CD3^+^CD4^+^ helper/inducer T cells, CD3^+^CD8^+^ cytotoxic T cells, CD3^−^CD19^+^ B cells, and CD3^−^CD16^+^, and/or CD56^+^ NK cells (Figure 6A). The results indicated that patients with enhanced CCL26^high^tumor cells had no significant differences in proportions and absolute numbers of CD3^+^ T cells, CD3^+^CD4^+^ helper/inducer T cells, CD3^+^CD8^+^ cytotoxic T cells, CD3^−^CD19^+^ B cells, and CD3^−^CD16^+^, and/or CD56^+^ NK cells in blood, and the phenomenon in CCL26^high^CAFs is similar (Figures 6B–E). This may be due to the fact that CCL26 acts in the local TIME (Chiu et al., 2017) and did not affect immune cells in the peripheral blood. In conclusion, OSCC with high expression of CCL26 was not associated with lymphocytes in the peripheral blood circulation.

## Discussion

CCL26 belongs to the CC chemokine subfamily, which consists of 27 chemokines that are essential for cell communication and tumor microenvironment regulation. Substantial studies have reported that numerous studies emphasize CCL26’s critical role in cancer biology and tumor immunity, notably, a unified conclusion regarding how CCL26 influences survival outcomes has not been reached in pan-cancer analysis (Uhlén et al., 2015; Uhlen et al., 2017). For instance, CCL26 overexpression correlates with poor prognosis in lung, liver, renal, and urothelial cancers, while in stomach, pancreatic, and endometrial cancers, higher levels are associated with longer overall survival. These variations suggest that CCL26’s biological role and impact on survival differ among cancers. Our prior research showed that CCL26, secreted by CAF^WPOI4-5^, promotes altered tumor invasiveness. CCL26 exhibited aberrant expression profiles in most solid cancers, which cause tumor microenvironment variation to influence tumor progression. While previous studies have touched upon the role of CCL26 in specific cancers, our investigation is the first to explore it in OSCC.

Previous studies have linked high CCL26 expression with poor prognosis across several cancers. However, in advanced melanoma, increased CCL26 levels have shown significant correlation with anti-PD1 antibody efficacy, suggesting potential benefits for these patients (Fujimura et al., 2020). Herein, we investigated the expression landscape of CCL26 in the TIME by exploring the expression details of diverse cell types and clinical prognosis. It is consistent with most previous studies, CCL26 was highly expressed in tumor cells and cafs and enrichment of these cells is associated with a trend toward decreased overall survival rate, showed a worse prognosis. Clinicopathological characterization showed that the expression of CCL26 in both cell types was significantly correlated with WPOI, which is an important prognostic indicator for local recurrence of OSCC. According a report, in colon cancer CCL26 is involved in tumor progression by regulating the EMT signaling pathway (Sun et al., 2022). Similarly, bioinformatics functional analysis showed that CCL26 make the mRNA expression of CLDN20/8, KRT36 etc., change which was demonstrated to be associated with EMT and pertinent to the key genes (E-cad, N-cad, VIM, SNAI2) involved in the EMT signaling pathway, aligning with previous studies. Our results support that CCL26 expression may play a prominent role in OSCC progression.

Recently, bioinformatics-based prognostic models incorporating CCL26 have been developed for various cancers, including esophageal adenocarcinoma (Zhang et al., 2021), hepatocellular carcinoma (Hu et al., 2021) and OSCC (Zhao et al., 2021) have been constructed based on bioinformatics technology. We explored CCL26’s molecular mechanisms in tumors using GSEA and GO analyses, which revealed that low CCL26 expression activates immune-related pathways. Previous study reported that in Hepatocellular carcinoma, hypoxia-induced MDSC infiltration is dependent on CCL26, it was profoundly promoting angiogenesis, and tumor growth (Chiu et al., 2016). Therefore, to better understand how CCL26 influences the OSCC tumor immune microenvironment, we turn to investigate immune checkpoint and immune cell infiltration molecules. In contrast to earlier studies, our results indicate a negative correlation between CCL26 and various immune cells, including T cells, B cells, NK cells, DC cells, and neutrophils. Another report showed that interaction of CCL26 and CCR3 regulates the Th2-dominant tumor environment. Here, we finally examined the major immune cells (T cells, B cells and NK cells) in peripheral blood by flow cytometry and found no effect on their numbers and percentages. The discussion suggests a potential link between CCL26 and the formation of a local immunosuppressive microenvironment, which may reduce immunotherapeutic efficacy by promoting cold tumors. Furthermore, the therapeutic approach of remodeling the immune microenvironment by transforming cold tumors into hot tumors has been proposed (Ishizuka et al., 2019; Jiang et al., 2019; Djajawi et al., 2024; Covre et al., 2015; Huang et al., 2024; Guirguis et al., 2023; Stone et al., 2017), which also provides ideas for immunotherapy of OSCC.

In summary, our findings provide valuable insights into the clinical distribution, prognostic and pathological features of CCL26 in OSCC. Importantly, we also revealed the expression details of CCL26 in TIME in OSCC for the first time. At the same time, offering a preliminary analysis of its association with immune cell infiltration, immune pathways, and classical immunotherapeutic markers, proposing it as a promising therapeutic target. Our results lack of the sufficient sample, this study shows a tendency to correlate with poor prognosis, but did not show the exact impact on the prognosis of oral cancer. Similarly, while our bioinformatics approach has revealed these associations, further basic and clinical studies are essential to fully elucidate the mechanisms of CCL26 in OSCC cytological behavioral function and immunity. Despite its preliminary character, it is becoming increasingly clear that analyzing the impact of CCL26 expression in OSCC may help to elucidate its role as a potential poor prognostic marker and help to identify possible clinically targeted therapies.

## Data Availability

The original contributions presented in the study are included in the article/Supplementary Material, further inquiries can be directed to the corresponding authors.
